# Endoscopic Management of Recurrent Acute Pancreatitis

**DOI:** 10.3390/jcm14072150

**Published:** 2025-03-21

**Authors:** Pier Alberto Testoni, Sabrina Testoni

**Affiliations:** 1 Gastroenterology and Gastrointestinal Endoscopy, La Madonnina Clinic, Vita-Salute San Raffaele University, 20100 Milan, Italy; 2 Unit of Gastroenterology and Gastrointestinal Endoscopy, IRCCS Policlinico San Donato, Vita-Salute San Raffaele University, 20100 Milan, Italy; sabrinatestoni@hotmail.com

**Keywords:** recurrent acute pancreatitis, recurrent idiopathic acute pancreatitis, ERCP-guided therapy

## Abstract

This review aims to summarize the role of endoscopic therapy in the management and outcomes of recurrent acute pancreatitis (RAP). RAP is a clinical entity characterized by repeated episodes of acute pancreatitis in the setting of a normal gland or chronic pancreatitis (CP). The aetiology of RAP can be identified in about 70% of cases; for the remaining cases, the term “idiopathic” (IRAP) is used. However, advanced diagnostic techniques may reduce the percentage of IRAP to 10%. Recognized causes of RAP are gallstone disease, including microlithiasis and biliary sludge, sphincter of Oddi dysfunction (SOD), pancreatic ductal abnormalities (either congenital or acquired) interfering with pancreatic juice or bile outflow, genetic mutations, and alcohol consumption. SOD, as a clinical entity, was recently revised in the Rome IV consensus, which only recognized type 1 dysfunction as a true pathological condition, while type 2 SOD was defined as a suspected functional biliary sphincter disorder requiring the documentation of elevated basal sphincter pressure to be considered a true clinical entity and type 3 was abandoned as a diagnosis and considered functional pain. Endoscopic therapy by retrograde cholangiopancreatography (ERCP) and endoscopic ultrasound (EUS) has been proven effective when a mechanical obstruction is found and can be removed. If an obstruction is not documented, few treatment options are available to prevent the recurrence of pancreatitis and progression toward chronic disease. In gallstone disease, endoscopic biliary sphincterotomy (EBS) is effective when a dilated common bile duct or biliary sludge/microlithiasis is documented. In type 1 SOD, biliary or dual sphincterotomy is generally successful, while in type 2 SOD, endotherapy should be reserved for patients with documented sphincter dysfunction. However, in recent years, doubts have been expressed about the real efficacy of sphincterotomy in this setting. When sphincter dysfunction is not confirmed, endotherapy should be discouraged. In pancreas divisum (PD), minor papilla sphincterotomy is effective when there is a dilated dorsal duct, and the success rate is the highest in RAP patients. In the presence of obstructive conditions of the main pancreatic duct, pancreatic endotherapy is generally successful if RAP depends on intraductal hypertension. However, despite the efficacy of endotherapy, progression toward CP has been shown in some of these patients, mainly in the presence of PD, very likely depending on underlying genetic mutations. In patients with IRAP, the real utility of endotherapy still remains unclear; this is because several unknown factors may play a role in the disease, and data on outcomes are few, frequently contradictory or uncontrolled, and, in general, limited to a short period of time.

## 1. Introduction

In 2018, recurrent acute pancreatitis (RAP) was defined by an international state-of-the-science conference as “a syndrome of multiple distinct acute inflammatory responses originating within the pancreas in individuals with genetic, environmental, traumatic, morphologic, metabolic, biologic, and/or other risk factors, who experienced 2 or more episodes of acute pancreatitis, separated by at least 3 months” [1]. RAP may occur in a normal pancreas or in chronic pancreatitis (CP). When minor ductal or parenchymal lesions are found in the gland, it is still an unsettled issue whether these lesions are the consequence of recurrent episodes of pancreatitis in an originally normal pancreas or occur in CP at an early stage. In fact, AP, RAP, and CP are now believed to lie on a continuous spectrum of disease.

Evolution from a morphologically normal pancreas toward CP has been reported to occur in 4% to 36% of RAP cases [2,3,4,5,6,7], with one study finding that out of all patients who had a fourth episode of RAP, 50% went on to develop CP [8]. Two meta-analyses documented that 10% and 36% of patients, respectively, developed chronic disease over the years after a first episode of AP and RAP [7] and that the occurrence of RAP lead to a threefold higher increase in the risk for developing CP after the first AP episode [9]. In a follow-up study on a large population, the probability of developing AP after a single attack of AP was 13% over 10 years and 16% over 20 years, but it increased to 36% within 2 years after the second attack [2]. The risk of progression toward CP is not the same in RAP patients because the etiology of pancreatitis plays a different role in the likelihood of progression to CP. Alcohol, smoking, pancreas divisum (PD), and genetic mutations are very likely involved in this progression.

In two studies, heavy alcohol consumption was associated with a risk of developing RAP and progressing toward CP in 58% and 41% of cases, respectively, with risk reduction to 20% and 13% with abstinence [3] and a significant risk of developing CP (OR 3.10) when compared to abstainers and light drinkers (<5 drinks/day) [10]. In a series of patients with heavy alcohol consumption, CP occurred in about 80% of cases over a 15-year follow-up [11]. Hegyi et al. reported that the rate of alcoholic etiology increased from AP to RAP to CP (19.4%, 39.1%, and 51.6%, respectively) [8].

Smoking has been considered a significant dose-dependent risk factor for RAP recurrence and evolution toward CP, too, but the benefits of smoking cessation have yet to be fully clarified [10]. Nøjgaard et al. found that tobacco smoking was the strongest risk factor associated with progression to CP [4]; however, in a population-based study, CP was associated with alcohol, unknown causes, gallstone disease, and smoking in 28%, 10%, 6%, and 1% of cases, respectively [5].

Mutations of the CFTR, SPINK 1, and PRSS 1 genes have been documented in RAP and CP [12,13,14,15,16,17,18,19]. Hereditary pancreatitis has the greatest risk of developing into CP, documented in about 50% of cases [18]. Genetic mutations have also been supposed to have a cumulative effect on the development of CP in symptomatic PD patients, even after successful endoscopic treatment [20,21,22]. In fact, an underlying chronic disease that made endoscopic therapy ineffective was reported in 47% of patients with non-alcoholic and non-biliary RAP over an 8-year follow-up period [23].

## 2. Endoscopic Management of Recurrent Acute Pancreatitis

Few effective therapeutic options are available for patients suffering from RAP; therapeutic endoscopy of the pancreatico-biliary ductal system (ERCP) is the most important one. The efficacy of ERCP-guided therapy depends on two main variables: the presence of a mechanical obstruction that can be removed and an underlying normal pancreas or chronic pancreatitis.

The aetiology of RAP is summarized in the TIGAR-O classification, so called due to being an acronym of the main predisposing risk factors (toxic–metabolic, idiopathic, genetic, autoimmune, recurrent and severe acute pancreatitis, obstructive) [24]. These risk factors can be identified in about 70% of patients, while in the others, the aetiology remains unknown, and these cases are defined as “idiopathic” (IRAP). However, in recent years, advanced diagnostic investigations have reduced the percentage of “idiopathic” cases to approximately 10% [25].

Among the causes of RAP, only those that determine pancreatic juice outflow obstruction can be effectively eliminated by therapeutic ERCP. Obstructions may depend on gallstone disease (including sludge and bile crystals), sphincter of Oddi dysfunction (SOD), PD, anatomical variants of the pancreatico-biliary junction, choledochoceles, and strictures of the main pancreatic duct (MPD), either benign or malignant. An obstruction with intraductal hypertension may also occur in RAP in patients with CFTR gene mutations, because of the density of pancreatic juice, and in those with hereditary pancreatitis with MPD strictures. However, evidence that endoscopic decompressive therapy improves the course of these diseases is still lacking.

Therapeutic ERCP includes sphincterotomy of the biliary (EBS) and/or pancreatic (EPS) segment of the sphincter of Oddi, minor papilla sphincterotomy, and pancreatic trans-papillary stenting to dilate MPD strictures and decompress the ductal system upstream of strictures or drain fluid collections.

## 3. Endotherapy for Recurrent Acute Pancreatitis of Biliary Etiology

Gallstone disease accounts for up to one-third of cases of RAP in Western countries [26]. In about 70% of these cases, RAP is caused by microlithiasis (stones ≤ 3 mm in diameter and biliary sludge) rather than common bile duct (CBD) stones [27], which have been reported in 4–24% of patients followed up for up to 15 years after cholecystectomy. A common channel at the pancreatico-biliary junction is an additional risk factor for RAP in gallstone disease because it may facilitate the reflux of bile into the pancreatic ducts.

Cholecystectomy is generally effective in treating RAP with a documented biliary etiology, even though a meta-analysis including 57,815 patients from 42 studies showed a pooled risk of 6.6% of recurrent AP after cholecystectomy following gallstone pancreatitis [28].

Empirical cholecystectomy has also been proposed when stones or sludge are not found, but the indication in these cases remains controversial [29].

EBS is the intervention of choice in RAP of biliary etiology when pancreatitis recurs after cholecystectomy. EBS can also be proposed as the first therapeutic step, instead of cholecystectomy, when CBD stones/microlithiasis are documented; in these cases, the gallbladder “in situ” has not been found to be a significant risk factor for complications after EBS, although this was hypothesized. Recurrence of pancreatitis was reported in 18% of cases after EBS with gallbladder “in situ”, while no recurrences were reported after both cholecystectomy and EBS [30]. Endoscopic papillary balloon dilation (EPBD) may be used as an alternative to EBS to remove CBD stones. This technique was found to be associated with lower complication and stone recurrence rates compared to EBS (5.3% vs. 17.3% and 4.4% vs. 12.7 for stones ≤ 8 mm) [31]. The incidence of post-ERCP pancreatitis (PEP) did not differ after EPBT and EBS in most studies assessing this [32]. EPBD is not indicated when CBD stones are not found or SOD is suspected.

## 4. Endotherapy for Recurrent Acute Pancreatitis Associated with Sphincter of Oddi Dysfunction

Although, in the past, SOD was considered to account for 35–65% of cases of RAP [33,34,35,36,37], in recent years, it has been critically reviewed both as a clinical entity and in terms of its role in the occurrence of AP and the efficacy of endoscopic sphincterotomy in preventing the recurrence of AP. However, in patients with RAP and CBD stones, significantly higher basal sphincter pressure gradients between the CBD and duodenum were documented, suggesting a possible primary role of sphincter dysfunction in the occurrence of pancreatitis [37].

SOD is characterized by either a persistent or transient increase in basal sphincter pressure and has been originally grouped into three types based on clinical, morphological, and functional parameters [38,39,40]. Sphincter dysfunction can involve either the biliary or pancreatic segment, or both. Consensual sphincter involvement is the most frequent condition. In a large series of patients with type 2 SOD, the consensual involvement of both sphincters, pancreatic sphincter only, or biliary sphincter only was reported in 32%, 22%, and 11% of cases, respectively [41]. While type 1 SOD (classified based on bile/pancreatic duct dilation and abnormal laboratory findings) represents a true pathological condition leading to a persistent obstruction, doubts have been raised about the clinical importance of types 2 and 3 SOD. In the Rome IV consensus, type 3 SOD was abandoned as a diagnosis and considered functional pain, while type 2 SOD was recommended to be termed suspected functional biliary sphincter disorder [42], requiring the documentation of elevated basal sphincter pressure to be considered a true clinical entity.

Endoscopic sphincterotomy is currently the standard non-pharmacological therapy for SOD. In general, EBS is performed first; if pancreatitis still recurs, EPS may be considered. Pancreatic sphincter hypertension was reported in up to 78% of patients with unsuccessful EBS [43]. Considering the high probability of consensual sphincter dysfunction, dual sphincterotomy (consensual EBS and EPS) is widely used to treat SOD [44,45].

In type 1 SOD, EBS alone has been reported to achieve clinical improvement in 83–100% of cases. More uncertain are the outcomes in type 2 SOD. In the presence of a documented sphincter dysfunction, EBS has been reported to be effective in up to 80% of cases, too.

In this regard, it should be emphasized that the documentation of an abnormal sphincteric basal pressure in type 2 SOD may be difficult in clinical practice because SOM has been mostly abandoned due to its invasiveness and poor diagnostic reliability and the secretin test is not available in most centers [46,47]. However, despite the encouraging results published years ago, in recent studies on large series of patients, the percentage of recurrent AP was reported to vary from 17.4% to 40% of cases in both groups [48,49]. In these cases, sphincter dysfunction was probably not the cause of RAP, although it was documented.

In type 2 SOD with an uncertain documentation of sphincter dysfunction and type 3 SOD, endoscopic sphincterotomy should not be recommended because, in these cases, the outcomes that were reported in large multicenter studies were substantially like those of controls or sham sphincterotomy patients [48,49,50], so the risks and benefits of the intervention must be carefully weighed up in these patients. Moreover, the risk of post-ERCP pancreatitis is higher than in gallstone disease [51]. In patients with RAP associated with pancreatic SOD, EBS or EPS alone and dual sphincterotomy showed similar rates of recurrent pancreatitis (64.7% and 76.9%), with a tendency toward higher rates in patients undergoing dual sphincterotomy during the first 12 months of follow-up [44]. These findings suggest that EPS may have been inadequate or else that there was a high incidence of re-stenosis. In a large retrospective study, 41.7% of patients undergoing EPS for RAP or pancreatic-type SOD required a re-intervention on the pancreatic sphincter because of re-stenosis [47]. RAP may also recur after successful EPS because pancreatic SOD may just be an epiphenomenon and not the primary cause of the disease. This hypothesis is further borne out by evidence showing that EPS in patients with SPINK1 gene mutations and SOD gave a poor symptomatic response [52], suggesting the presence of an underlying early-stage CP.

These data were further confirmed in the Cochrane Database of Systematic Reviews published in 2024; the analysis of the available randomized clinical trials raised concerns about the clinical efficacy of endoscopic sphincteroromy in adults with biliary SOD. Based on the trials included in this review, evidence that EBS/EPS versus sham or versus dual sphincteroromy increase, reduce, or make no difference in the number of people with treatment success was lacking because of the low certainty of evidence for all the outcomes [53].

## 5. Endotherapy for Recurrent Acute Pancreatitis in Pancreas Divisum and Anomalous Pancreatico-Biliary Junction

PD and an abnormally long common pancreatico-biliary channel without sphincters separating the biliary and pancreatic duct are the most common anatomical variants of the pancreas associated with RAP. PD has been reported in about 20% of patients with RAP [54,55,56,57].

The inability of the minor papilla to accommodate pancreatic juice outflow results in a persistent or transient intraductal hypertension that may lead to RAP or obstructive CP in some patients. Endoscopic and surgical therapy in the management of PD are comparably effective in 70–90% of cases [56,57]; however, endotherapy is preferred, even though a meta-analysis reported higher rates of clinical success (72% vs. 62.3%) and fewer complications (23.8% vs. 31.3%) and re-interventions (14.4% vs. 28.3%) after surgery [58]. Three recent meta-analyses and a Dutch cohort study showed that the role of endoscopic therapy is variable but achieves the highest success rates in RAP patients compared with CP or pancreatic pain alone, with pooled reported healing rates of 69.8%, 71%, 76%, and 44.4% [59,60,61,62]. However, in the Dutch study, RAP patients who did not completely respond to treatment had a significant decrease in the mean number of AP episodes and interval between each episode after treatment [62].

Endoscopic therapy includes minor papilla sphincterotomy and/or stenting or dilation and is indicated when there is a dilated dorsal duct. This finding suggests the presence of some papillary obstruction and predicts a positive outcome after sphincterotomy or stenting. In the presence of a non-dilated dorsal duct, outcomes of endotherapy are uncertain, so the decision to perform minor papilla sphincterotomy should be guided by the documentation of some impairment of juice outflow through the minor papilla. After sphincterotomy, dorsal duct stenting for a short period is recommended to avoid post-ERCP pancreatitis and cicatricial strictures. Balloon dilation with stent placement was reported as an alternative technique to sphincterotomy in one study including several RAP cases, with a 100% technical success rate and an 85.7% rate of clinical improvement. Early complications were observed in 6.3% of patients, but post-ERCP pancreatitis or bleeding related to balloon dilation were not observed [63]. Stenosis after sphincter section occurs in 20–30% of cases and accounts for recurrent episodes of pancreatitis after endotherapy [56,63]; in these cases, a revision of the papillotomy and/or long-term pancreatic stenting (7 F to 10 F, depending on the dorsal duct diameter), for up to 12 months with stent exchange every 3 months, may be effective [64,65].

Even after a successful endotherapy, a tendency towards progression to CP may persist in some patients, very likely depending on underlying genetic mutations. CFTR gene and SPINK 1 gene mutations were documented with a higher frequency in patients with symptomatic PD compared with those with idiopathic or alcoholic pancreatitis and healthy controls [21,22,66] and may play some role in RAP and progression toward CP. In our series of PD patients suffering from RAP, endoscopic ultrasound (EUS) findings consistent with CP were seen in similar percentages in patients undergoing endotherapy and in the observation group (63.2% and 57.1%) over a five-year period, though endotherapy abolished episodes of AP in most cases [20].

In patients without dorsal duct dilation, minor papilla sphincterotomy is not indicated because an obstructive cause is not documented. In these cases, empirical dorsal duct stenting (up to 3 months and repeated if effective) may identify some unrecognized sphincter malfunction and helps predict which patients could benefit from sphincterotomy. In the only randomized controlled trial comparing dorsal duct stenting with no therapy in PD patients with RAP and a non-dilated dorsal duct, RAP episodes were significantly lower in the stent group than in the control group [58].

An abnormally long (>15 mm) common pancreatico-biliary channel is an anatomical condition that facilitates the reflux of bile into the pancreatic ductal system and may be associated with RAP. In this condition, EBS reduces the intra-ampullary resistance to pancreatic juice outflow and the risk of pancreatitis [67]. However, the role of endoscopic therapy is limited, because it is not effective when the anomalous junction extension goes beyond the anatomic limit of biliary sphincterotomy [68].

## 6. Endotherapy for Recurrent Acute Pancreatitis in Chronic Pancreatitis and Acquired Obstructive Conditions

RAP superimposed on CP can occur due to intraductal hypertension caused by stenosis of the pancreatic sphincter, MPD strictures, or pancreatic intraductal stones. MPD strictures occur in 5–10% of cases of RAP. Fluid collections may occur even in mild-change CP and can promote further episodes of pancreatitis, too, if not resolved. Segmental MPD strictures may also depend on residual scars after an episode of severe acute pancreatitis, pancreatic trauma, or neoplastic conditions. In the absence of morphological findings suggestive for chronic pancreatitis, the differential diagnosis between a benign and malignant nature of the stricture is pivotal before planning any treatment. EPS, dilation, and stenting are the mainstays of endoscopic therapy [69].

EPS can be either a dual-step (EBS first, followed by EPS) or a single-step (direct EPS) procedure [31]. Dual-step sphincterotomy gives a better visualization of the anatomy prior to EPS, making sphincter section technically easier and more precise. However, data to support this hypothetical advantage are lacking. More importantly, consensual EBS and EPS were associated with a lower risk of cholangitis [70,71,72].

EPS as a single therapeutic intervention has been reported to be effective in the presence of stenosis of the pancreatic sphincter and when MPD is dilated without strictures. In CP, these findings are mainly seen in the disease with mild-to-moderate ductal changes, according to the Cambridge classification [73]. However, few data on outcomes are available; in two series of CP patients undergoing therapeutic ERCP, detailed outcomes in those with mild-change disease were not reported [74,75]. The most robust predictor of a successful long-term outcome after EPS is the location of the obstruction proximal to the papillary orifice. The absence of dominant MPD strictures and intraductal stones, together with a small number of RAP episodes before endotherapy, are other predictors of good outcomes [76].

Trans-papillary stenting is the intervention of choice in dominant MPD strictures (ductal dilation of ≥6 mm in diameter upstream of the stricture) not involving the papillary orifice. Stenting may or may not be associated with EPS. It decompresses the MPD and achieves a persistent dilation of the stricture if the stent is maintained for up to 24 months, with 3-/6-month stent exchange. Pain relief and cessation of RAP were reported in 51.5–80.2% of patients after long-term stenting [72]. The insertion of a single 10 F plastic stent is the standard first approach in the treatment of MPD strictures [77]; however, in mild-to-moderate-ductal changes, 7 F/8.5 F stents could be more appropriate because the diameter of the stent should not exceed that of the MPD. Small-diameter stents may expose patients to more frequent recurrencies of symptoms; in a retrospective study, ≤8.5 F stents were 3.2 times more likely to be associated with hospitalization than 10 F stents [78]. In the presence of tight strictures, the insertion of a 10 F plastic stent may require mechanical or pneumatic dilation. In refractory strictures (symptomatic strictures that persist or relapse after 1 year of single pancreatic stent insertion), multiple side-by-side plastic stents or fully covered self-expandable metal stents (FCSEMSs) can be inserted [79]. The insertion of multiple side-by-side plastic stents was reported to resolve strictures in about 90% of patients, over a 9.5-year follow-up [80,81], but a recent study reported a recurrence of pain after stent removal in 42% of patients [82]. For refractory strictures in the head of the pancreas, FCSEMSs (6–10 mm in diameter) are an effective alternative to multiple plastic stenting. In a recent systematic review, the pooled stricture resolution rate with FCSEMSs was 91.6%, while the pooled pain/recurrent pancreatitis resolution rate was 84.9%. The pooled incidences of adverse events, including AP, pain requiring stent removal, and de novo stricture, were 3.9%, 0.8%, and 3.3%, respectively. The rates of stent migration, stricture recurrence, and the need for restenting were 12.9%, 9.3%, and 12.3%, respectively [83]. FCSEMSs can stay in place for longer than plastic stents and are generally changed every 2–6 months, with uneventful removal in 98% of patients [83]. Biodegradable non-covered self-expandable stents are currently being investigated for the treatment of MPD strictures. In a pilot study, this type of stent was clinically successful in 53% of patients in whom 6-month plastic stenting had failed, with adverse events reported in 21% of patients [84]. However, the available data do not support the use of these stents outside clinical trials. In the case of unsuccessful ERCP-guided pancreatic stenting, EUS-guided MPD drainage represents an effective emerging alternative treatment for obstructive CP, but the high incidence of adverse events associated with this technique (approximately 20%) remains an issue. A recent paper reported a pilot study on a novel plastic stent for EUS-guided pancreatic drainage, with technical and clinical success rates of 100% and no complications [85].

In CP, fluid collections located upstream of an MPD stricture or larger more than 5 cm rarely regress spontaneously. Two factors contribute to this complication: sustained intraductal hypertension and communication between the ductal system and the fluid collection itself. Small collections communicating with the MPD can be drained by an ERCP-guided approach as an alternative to EUS-guided transmural drainage. Trans-papillary MPD stenting allows the cavity to drain by inverting the pressure gradients between the ductal system and duodenum. The stent can also be used to bridge the communication between the MPD and fluid collection to prevent the fluid from entering the cavity. Stent insertion is easier when the fluid collections are in the head or body of the pancreas and is successful in 33–67% of patients. Double-pigtail stents should be preferred to straight ones because of the lower risk of displacement and parietal damage inside the cavity. EUS-guided transmural drainage can be used if trans-papillary drainage fails or as a first-choice procedure in the presence of large fluid collections [86,87]. Compared with trans-papillary drainage, EUS-guided transmural drainage achieves similar success with similar morbidity rates but more complications requiring surgery [88]. The benefit of adding trans-papillary drainage to transmural EUS-guided fluid collection in the presence of a disrupted MPD is controversial and probably absent, as documented in a meta-analysis [89].

**Figure 1:**
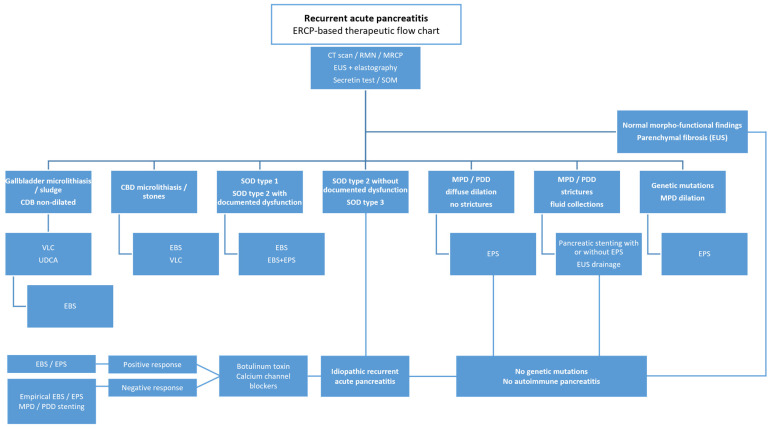
MRCP = magnetic resonance cholangiopancreatography; EUS = endoscopic ultrasound; SOM = sphincter of Oddi manometry; ERCP = endoscopic retrograde cholangiopancreatography; CBD = common bile duct; MPD = main pancreatic duct; PDD = pancreatic dorsal duct; SOD = sphincter of Oddi dysfunction; VLC = video-laparoscopic cholecystectomy; EBS = endoscopic biliary sphincterotomy; EPS = endoscopic pancreatic sphincterotomy; UDCA = ursodeoxycholic acid.

A flow chart of endoscopic therapy in RAP is reported in Figure 1.

## 7. Endotherapy for Recurrent Acute Pancreatitis in Rare Obstructive Conditions

Other acquired obstructive conditions that can induce RAP are ampullary neoplasms, intraductal papillary mucinous neoplasia (IPMN), and periampullary diverticula (PADs). Rare conditions are choledochoceles and ampullary choledochal cysts.

Ampullary adenomas or carcinomas confined within the muscolaris mucosae and not involving the pancreatico-biliary ductal system can be resected endoscopically after EUS staging. The lesion can be resected en bloc or piecemeal by snare papillectomy. En bloc resection without mucosal lifting can be used when the neoplasia is confined within the ampulla; piecemeal resection with mucosal lifting should be performed in the presence of residual adenomatous tissue around the papilla [90].

IPMN causes RAP because the abnormal mucin secretion produces a dense juice that induces intraductal hypertension. Pancreatitis has been reported to occur more frequently in the main-duct/combined type than in the branch-duct type (14% vs. 5%) [91]. Dual sphincterotomy facilitates juice outflow through the papilla; complete and partial responses (>50% reduction in AP episodes) were reported in 64% and 24% of cases, respectively, with an overall response rate of 88%, in a mean follow-up of 93.4 months [92].

It is still unclear whether PADs are directly involved in the recurrence of pancreatitis, although these diverticula are frequently found in both biliary pancreatitis and RAP in middle-aged subjects. There are several purported mechanisms by which PADs at or near the ampulla of Vater are implicated in the pathogenesis of RAP: direct obstruction of pancreatic juice/bile outflow, higher occurrence or recurrence of bile duct stones, and possible association with SOD causing transient hypertension or biliary reflux into the pancreatic ductal system. Treatment guidelines for PADs are not currently available but an ERCP-guided approach is commonly carried out. Endoscopic treatment may be difficult due to the risk of perforating the diverticulum and hemorrhage, so a wire-guided EBS or EPBD should be preferred; if RAP still occurs, EPS could be considered. However, the reported success rates are variable.

Choledochoceles are an anatomical condition in which the intramural segment of the CBD is dilated and herniates into the duodenal lumen; biliary sludge/stones in the dilated ampulla or cystic dilation per se may obstruct the outflow of pancreatic juice and favor episodes of acute pancreatitis. Endoscopic section of the bulging part of the CBD by sphincterotome or needle-knife is usually effective.

## 8. Endotherapy for Idiopathic Recurrent Acute Pancreatitis

In IRAP, there is limited evidence that endoscopic therapy can really affect the course of the disease and prevent the progression toward CP [68,93,94,95,96]. In the North American Pancreatitis Study 2 (NAPS 2), the rates/year of pancreatitis recurrence did not differ between IRAP patients who had undergone endotherapy and those who had been managed conservatively, and progression to CP was more frequent in the former than the latter (27% vs. 8%) [96]. Another follow-up study reported progression toward CP in 20–50% of cases, independently from the efficacy of endotherapy [44]. Overall, these data confirm that in a high percentage of cases suffering from IRAP, there is an underlying chronic pancreatic disease that may render endoscopic treatment ineffective.

Occult gallstone disease and some unrecognized SODs are very likely the most common causes of IRAP in adults. Biliary crystals have been detected in up to 75% of patients and are considered the primary cause of disease in 22% [43,97], as documented by the efficacy of ursodeoxycholic acid (UDCA) therapy, cholecystectomy, or EBS in preventing further episodes of pancreatitis [27,35,43,98]. A meta-analysis carried out by the Dutch Pancreatitis Study Group in 524 patients with IRAP documented a reduction in the recurrence of AP from 35.2% to 11.1% following cholecystectomy [99].

Empirical EBS could be considered when a previous cholecystectomy failed to prevent recurrencies in IRAP. In a recent study, AP recurred in 46% and 18.8% of patients after cholecystectomy and EBS, respectively [97]. Empirical sphincterotomy was found to be effective in 87.5% of IRAP patients in our experience, too [98]. However, the RESPOnD study reported a 31% recurrence rate of AP after sphincterotomy (either EBS or EPS or dual) in these patients [49], like an earlier randomized trial [44]. These rates are worse than those reported (15.1%) in a meta-analysis of 17 studies and 4754 individuals with IRAP [28]. In fact, consensus guidelines on empirical sphincterotomy in IRAP patients are not definitive in their recommendations and it remains unclear to what extent this option is being used in clinical practice [1], so in these cases, as for sphincterotomy in suspected SOD with unrecognized sphincter dysfunction, the decision to perform some endoscopic therapy should be discussed with the patient, considering the potential risks and unpredictable outcomes of this intervention.

As an alternative to EPS, empirical pancreatic stenting could be considered to predict the response to EPS, but few reports are available so far. We used 5 F and 7 F pancreatic plastic stents, after informed discussion with patients, with a significant reduction in pancreatitis episodes and subsequent successful outcomes after EPS [98]. In another prospective study including 34 IRAP patients randomized to receive pancreatic stents over 9 months, with stent exchange every 3 months, the incidence of RAP was significantly lower in the stent group compared to the control group (11% vs. 53%) [100]. The efficacy of pancreatic stenting in IRAP was recently confirmed in a pediatric population with a 24-month follow-up after stent withdrawal, with resolution of episodes of pain and pancreatitis in all patients and with mild complications in 15.7% of cases [101]. These results confirm that some unrecognized MPD hypertension may account for the recurrence of pancreatitis in a subset of IRAP patients with normal-appearing pancreas. However, there are so far insufficient data in the literature to include empiric MPD stenting as a diagnostic tool in IRAP. Moreover, stenting a non-dilated pancreatic ductal system, even for a short time, may cause ductal injury mimicking CP. Whether these changes may or may not regress once the stent is removed is still an unsettled issue [102].

As an alternative to empirical sphincterotomy or stenting, botulin toxin injection into the papilla of Vater may help identify some sphincter malfunction. A positive response to botulinum toxin injection was reported in 80% of cases with suspected SOD and non-dilated ducts and was shown to predict which patients were most likely to improve with EBS/EPS [103]. The effect of botulin toxin is transient, so it is useless in patients in whom episodes of pancreatitis occur at intervals greater than 3–4 months; moreover, there is some concern that repetitive injections may cause fibrosis of the sphincter. Botulin toxin can also be injected into the minor papilla in IRAP occurring in PD with non-dilated dorsal duct [104]. However, botulinum toxin injection remains, for now, an experimental treatment modality with little evidence. Calcium channel blockers (nifedipine, nicardipine) and glyceryl trinitrate have also been used in suspected SOD to treat biliary pain, but no studies on the prevention of pancreatitis have been published so far.

A flow chart of endoscopic therapy in IRAP is reported in Figure 1.

## 9. Conclusions

ERCP-guided endotherapy in RAP patients has been proven effective in gallstone disease, type 1 SOD, and in the presence of any obstructive condition, either congenital or acquired, associated with a dilation of the pancreatic ductal system. In type 2 SOD, the role of endotherapy remains controversial and should be reserved for patients with documented sphincter dysfunction, while for type 2 SOD without documentation of sphincter dysfunction and type 3 SOD, doubts have been raised in recent years about the real efficacy of sphincterotomy; there is a critical need for randomized controlled clinical trials that provide high-level evidence, with unified classifications and standardized metrics for evaluation. In PD, minor papilla sphincterotomy is effective when a dilated dorsal duct is found, and the success rate is the highest in RAP patients.

Endoscopic therapy in IRAP remains challenging because morpho-functional findings appear to be normal, and outcomes are uncertain. Most studies in patients undergoing endotherapy have included both subjects with functional pain and recurrent pancreatitis, so it is difficult to draw up reliable data on this topic. Empirical cholecystectomy, EBS and subsequent EPS, and pancreatic stenting have been attempted, mainly in small-sample-size or retrospective studies, with success rates varying from 0 to approximately 50%. Botulinum toxin injection into the papilla of Vater has been found to help to identify patients in whom sphincterotomy may be successful, but few reports are available so far. Successful empirical sphincterotomy or stenting or a positive response to botulinum toxin confirm that unrecognized obstructive conditions may induce transient intraductal hypertension in a normal pancreas, leading to RAP. However, despite successful endotherapy, progression toward CP has been shown in up to 50% of these patients, very likely depending on other underlying unknown factors or genetic mutations.

In fact, in patients with IRAP, the real utility of endotherapy remains unclear, so we need to improve clinical investigations and promote further trials with high levels of evidence.
